# MEMS Membranes with Nanoscale Holes for Analytical Applications

**DOI:** 10.3390/membranes11020074

**Published:** 2021-01-20

**Authors:** Alvise Bagolini, Raffaele Correale, Antonino Picciotto, Maurizio Di Lorenzo, Marco Scapinello

**Affiliations:** 1 MNF, CMM, Fondazione Bruno Kessler, Via Sommarive 18, 38123 Trento, Italy; picciotto@fbk.eu; 2 Nanotech Analysis SRL, Corso Umberto 65, 10128 Torino, Italy; r.correale@nanotechanalysis.com (R.C.); m.dilorenzo@nanotechanalysis.com (M.D.L.); m.scapinello@nanotechanalysis.com (M.S.)

**Keywords:** MEMS, membrane, holes, mass spectrometer, low stress, silicon nitride, polysilicon

## Abstract

Micro-electro-mechanical membranes having nanoscale holes were developed, to be used as a nanofluidic sample inlet in novel analytical applications. Nanoscopic holes can be used as sampling points to enable a molecular flow regime, enhancing the performance and simplifying the layout of mass spectrometers and other analytical systems. To do this, the holes must be placed on membranes capable of consistently withstanding a pressure gradient of 1 bar. To achieve this goal, a membrane-in-membrane structure was adopted, where a larger and thicker membrane is microfabricated, and smaller sub-membranes are then realized in it. The nanoscopic holes are opened in the sub-membranes. Prototype devices were fabricated, having hole diameters from 300 to 600 nm, a membrane side of 80 μm, and a simulated maximum displacement of less than 150 nm under a 1 bar pressure gradient. The obtained prototypes were tested in a dedicated vacuum system, and a method to calculate the effective orifice diameter using gas flow measurements at different pressure gradients was implemented. The calculated diameters were in good agreement with the target diameter sizes. Micro-electro-mechanical technology was successfully used to develop a novel micromembrane with nanoscopic holes, and the fabricated prototypes were successfully used as a gas inlet in a vacuum system for mass spectrometry and other analytical systems.

## 1. Introduction

Nanoscale fluidics are key to enable a range of novel fluidic devices [1]. In recent years, nanoscale holes have been used as sample inlets for the development of high-performance spectrometer gas sensors, leveraging the combination of nano technologies with ultra-high vacuum (UHV) techniques.

There are solid reasons to operate at the nanoscale level for analytical purposes. It is well known that the different parts of a mass spectrometer (MS) can only operate in an environment where stringent vacuum conditions are satisfied. Gas analyzers (GAs), multi gas analyzers, and in specific cases, gas chromatograph mass spectrometers (GC-MS) can operate from a few mbar up to atmospheric pressure to monitor processes in the semiconductors industry, refrigeration industry, and in leak detection fields. Such GAs are submitted to the typical MS vacuum constraints, which strongly depend on inlet sample flows and vacuum systems. A great improvement in system simplification [2,3,4,5] is possible by using nanometer-scale orifices [5] as sampling points and smart gas interfaces toward atmospheric pressure.

At high pressure gradients, orifices with diameters of about 100 nm operate in a molecular flow regime (MFR) or close to it [6]. A flow through conductance in an MFR does not give origin to gas collective motions, preventing condensation effects, chemical reactions, and even clogging events [5]. Inlet gas flows in the range from 10^−5^ to 10^−7^ mbar L/s allow the realization of new GAs and GC-MS with an ultra-simplified vacuum system [2,3], without impacting the ultimate sensitivity [4,5]. By design, they guarantee the same concentrations of the external gases’ composition into the ionization chamber [7,8,9]. New families of innovative analytical instruments, which are portable and with high sensitivity, are thus made possible.

Such small orifices can be achieved using widely adopted silicon micro-nano fabrication processes, by etching nanoscale holes on a freestanding membrane. The membrane will be exposed to a 1 bar pressure gradient, having the sensing chamber on one side (in vacuum) and the analyte-containing ambient (at atmospheric pressure) on the other.

Silicon-microfabricated free-standing membranes have been developed for several applications ranging from pressure sensors [10] to microphones [11] and microheaters [12]. Among the commercially available microfabricated membranes, some different options are available. Perforated membranes capable of withstanding high pressure gradients (up to 25 bar) and having micron-size holes are produced by KTH [13]. In addition, 200 nm thick silicon nitride membranes are commercially available by Agar Scientific with holes down to 100 nm and having a side length of 500 μm [14]. Silicon-rich silicon nitride is one of the favorite materials for suspended membranes, as it has a mature and commercially available deposition process, e.g., [15], and low tensile residual stress, enabling a film thickness >150 nm. Nevertheless, there is no report in the literature of a microscopic membrane capable of withstanding a 1 bar pressure gradient and having nanoscopic holes on it, because of the following combined technological limitations: Membrane etching, membrane materials, and nanoscopic holes patterning.

Typically, membranes are realized on silicon wafers by removing silicon from the wafer backside with wet anisotropic etching [16] or deep reactive ion etching [17]. Wet etching is less versatile as it follows a crystal plane orientation, and may have a large footprint on the wafer backside. Deep reactive ion etching (DRIE) is more versatile but has a major limitation in the depth/width aspect ratio of silicon etching: The smaller the membrane side and the thicker the wafer, the harder it becomes to fabricate with DRIE. In industrial processes, a safe aspect ratio between the width and depth of the etched feature is assumed to be 10/1: Considering a wafer thickness of 625 μm (6 inch (15.24 cm) silicon SEMI standard wafer), the minimum membrane side would then be 62 μm.

Materials to be used for the fabrication of the freestanding membrane are limited by the need of a very low residual stress, possibly below 50 MPa [10,12]. A higher compressive residual stress may induce buckling, while higher tensile stress adds to the stress induced by the pressure load, making the membrane more fragile.

Holes can be opened on a freestanding MEMS membrane using microfabrication techniques. Nanoscale holes will require a planar surface to allow for proper patterning. In particular, photoresist spin coating poses limitations as thin (few 100 nm) photoresists are used for sub-micron patterning, which require a planar surface. As a rule of thumb, steps higher than the photoresist thickness on the surface may cause spin coating failure and poor process yield. Further, holes through the membrane film must be kept at a low aspect ratio, to avoid complex etching tuning and to have an optimal fluidic performance. Therefore, in the case of a 100 nm hole, the membrane film thickness has to be in the same size range as the hole width, which is a few hundred nanometers.

Combining nanoscale holes with a freestanding membrane therefore implies that the membrane thickness will be a few 100 nm, imposing a severe limitation on its ability to withstand a pressure gradient. This constraint must be combined with the above-mentioned limited materials choice, due to residual low-stress requirements. The maximum stress in a circular membrane under a pressure gradient is described by [18]:

$$S=\frac{3}{4}\cdot \frac{a^{2}}{t^{2}}\cdot P$$

where *a* and *t* are the membrane diameter and thickness, respectively, and *P* is the applied pressure. Considering a tensile strength of 1 GPa for silicon-rich silicon nitride [19], using a safety margin of 20% of the total stress and a design thickness of 200 nm, the resulting membrane diameter would be 22 μm, which, given the above considerations on safe DRIE aspect ratio, is challenging for DRIE microfabrication on 6 inch (15.24 cm) wafers, requiring an etching aspect ratio >30.

Further, due to the pressure gradient, a membrane with this thickness and side dimensions will have a center displacement of about 1 μm. This indicates that the working condition for such a membrane is a critical one, with a possible fault due to the mechanical wear generated by such a large displacement over time.

To overcome the above-mentioned limitations, in the present work, we report the development of freestanding MEMS membranes with nanoscale holes, using a novel membrane-in-membrane structure: A thick support membrane was designed, where smaller sub-membranes were fabricated, having nanoscopic holes. Finite element simulation was used to set the dimensions as a function of the materials properties. Based on simulation results, a batch of prototype devices was fabricated using micro-electromechanics (MEMS) technology on 6 inch (15.24 cm) SEMI standard silicon wafers.

Prototypes were packaged and tested with a dedicated setup to demonstrate their functionality. A method was reported to calculate the effective holes diameter by measuring the gas flow through the holes under different pressure gradients.

## 2. Materials and Methods

### 2.1. Device Fabrication Sequence and Simulation

**Figure 1:**
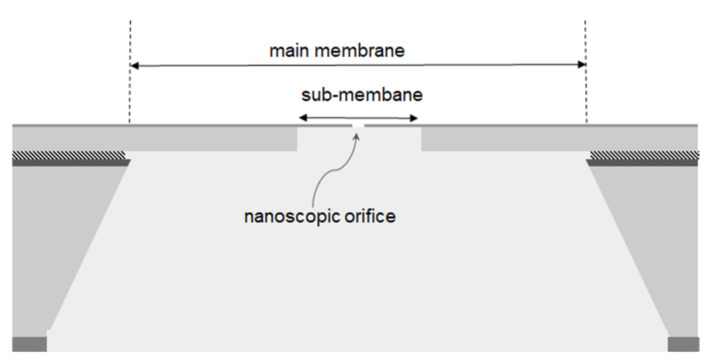
Membrane-in-membrane schematic.

To combine the advantages of a thick layer membrane (mechanical resistance to the pressure gradient) with those of a thin layer membrane (nanoscale holes patterning), a membrane-in-membrane structure was developed, as reported in Figure 1.

The material chosen for the thick membrane structure is polysilicon. It has a mature low-pressure chemical vapor deposition (LPCVD) process and can be tuned to near-zero residual stress by annealing at 1050 °C [20,21]. The yield stress of polysilicon is 3 GPa [22], and a thickness of up to 2 μm can be reached in a single deposition cycle. Moreover, polysilicon can be easily wet-etched using a well-assessed alkaline-based solvent process, to release the sub-membrane.

For the thin sub-membrane, silicon-rich silicon nitride was developed on a furnace model E1200HT by Centrotherm (Blaubeuren, Germany). The flow rates of dichlorosilane and ammonia were set to 160 and 16 sccm, respectively, obtaining a 10-to-1 ratio [23]. Three samples were deposited at different temperatures, with a deposition time of 100 min each. The deposition temperature was tuned to obtain a tensile residual stress below 50 MPa. Nitride stress was measured by the wafer curvature variation method using the Stoney Hoffman model [24]. The refractive index was measured by ellipsometry at 632.8 nm to account for the change in the material composition [23]. Data are reported in Table 1, including the deposition rate. Silicon nitride deposited at 790 °C was chosen as the best compromise for membrane fabrication, having a slightly tensile residual stress and an acceptable uniformity on a 25-wafer batch. The lowest stress was obtained by deposition at 795 °C, but this is a limit temperature for the furnace, and it causes poor uniformity because the temperature profile cannot be properly tuned at this temperature.

**Figure 2:**
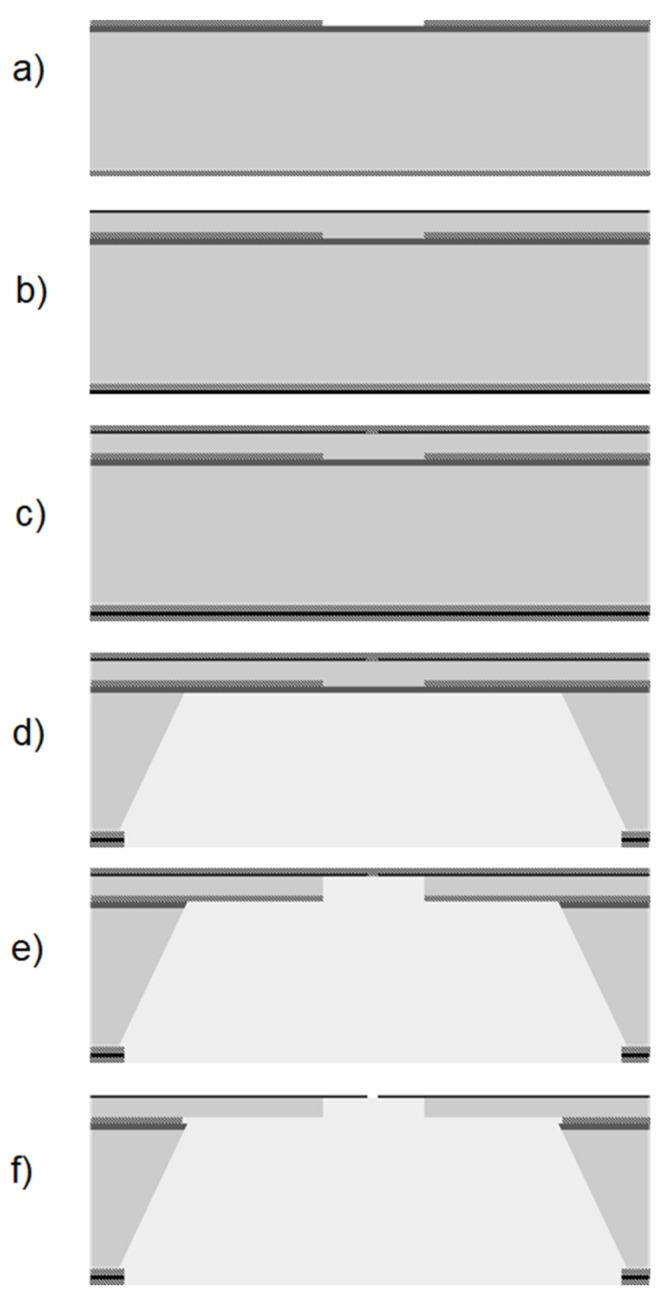
Membrane-in-membrane fabrication sequence schematic. Buried mask deposition and pattern (**a**), polysilicon and silicon-rich silicon nitride deposition (**b**), orifice patterning and silicon oxide deposition (**c**), backside mask pattern and anisotropic bulk etching (**d**), polysilicon etching from the wafer backside (**e**), and final orifice release by silicon oxide wet etching (**f**).

The developed fabrication sequence for the membrane-in-membrane devices is reported in Figure 2. The process is performed on SEMI standard 6 inch (15.24 cm) silicon wafers. All process steps are standard IC fabrication steps, the full details of which are reported in the Appendix A. A silicon nitride barrier for the final anisotropic etching is initially deposited by LPCVD, followed by LPCVD TEOS (tetraethoxysilane glass) deposition and patterning (Figure 2a). This glass mask will be used for the final etching of the polysilicon membrane from the wafer backside, to release the sub-membranes. As this glass mask is patterned at the beginning of the process and the device layers are then deposited on top of it, we refer to it as a buried mask. Next, polysilicon is deposited by PECVD at 620 °C, and recrystallized at 1050 °C for 30 min in a nitrogen atmosphere. PECVD silicon-rich silicon nitride is then deposited (Figure 2b). Nanoscale orifices are patterned in the silicon nitride layer, using the stepper photolithography technique, and a second TEOS is deposited to provide protection of the membranes during further fabrication steps (Figure 2c). Subsequently, the wafer backside mask is patterned, and anisotropic silicon etching is performed (Figure 2d) using tetramethylammonium hydroxide (TMAH). This etching releases the main membrane and determines its size. The etching geometry is governed by the anisotropy of TMAH etching along the 100 and 111 crystal planes. This anisotropy can be used to fine-tune the membrane size as it is related to the etching temperature. We measured the etching rate ratio between 100 and 111 crystal planes of 20/1 at 90 °C, which decreases to 12/1 at 80 °C. This provides a wide range for tuning the membrane side during anisotropic silicon etching.

The silicon nitride barrier for TMAH etching is then removed from the membrane backside (Figure 2e) and another TMAH etching is performed to remove the polysilicon and release the sub-membrane, using the TEOS buried mask. After dicing, the nanoscale orifices are finally freed by silicon oxide wet etching (Figure 2f).

The sub-membrane patterning is performed at the end of the process, using the buried silicon oxide mask as reported in Figure 2e: This allows for a flat surface on the front side, enabling nanoscale holes patterning with standard photolithography, as well as e-beam lithography techniques. TMAH is chosen to etch silicon from the backside because of DRIE limitations in aspect ratio, as mentioned above, and further because of wettability limitations in the DRIE-etched holes: Indeed, a liquid etchant solution must reach the membrane backside for silicon nitride etching, polysilicon etching, and silicon oxide final release, and this can be difficult in a high-aspect-ratio DRIE hole due to capillary forces.

**Figure 3:**
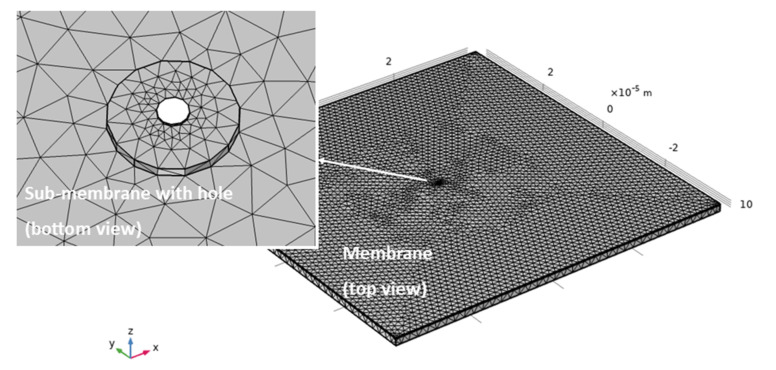
Model mesh of a 50 μm side membrane with a thickness of 1.7 μm, having a sub-membrane at its center, with radius of 2 μm and thickness of 200 nm, and an orifice diameter of 500 nm.

Finite element modeling was used to determine the membranes thickness as a function of the maximum displacement given by a 1 bar-pressure gradient. Ansys^TM^ software was used for this modeling. An image of the model mesh is reported in Figure 3.

Meshing was performed using tetrahedron elements, with element size refinement in the orifice area down to 100 nm in size. The membrane was constrained at the edges imposing a fixed support condition on the edge areas. A simulation was performed in static structural mode with linear elastic material behavior. The solution was calculated for stress (von-Mises equivalent) and total deformation. The preliminary simulation showed that the thickness of the silicon nitride layer is not influential on the overall mechanical performance of the membrane in the range from 100 to 300 nm, which is the range of interest for nanoscopic patterning. Therefore, the membrane side and the polysilicon thickness were considered as main parameters to determine the membrane response to 1 bar pressure gradient.

**Figure 4:**
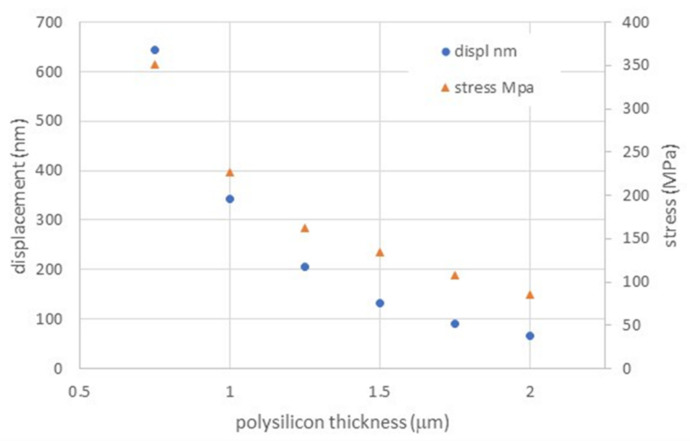
Maximum displacement at membrane center as a function of polysilicon thickness, with membrane side of 100 μm and a silicon nitride thickness of 200 nm.

Figure 4 shows the relation between maximum membrane displacement (at the membrane center) and polysilicon thickness. The maximum stress is also reported, showing that the membrane is always operating in a safe regime, far below 15% of the polysilicon 3 GPa tensile strength [23]. To obtain a compromise between processing time (polysilicon LPCVD) and mechanical performance, the polysilicon thickness was set at 1.5 μm, corresponding to a maximum simulated deflection of 133 nm and a maximum stress of 134 MPa. As can be seen from the simulation data, there is a large margin to tailor this structure to a range of performances while maintaining it in a safe stress regime.

**Figure 5:**
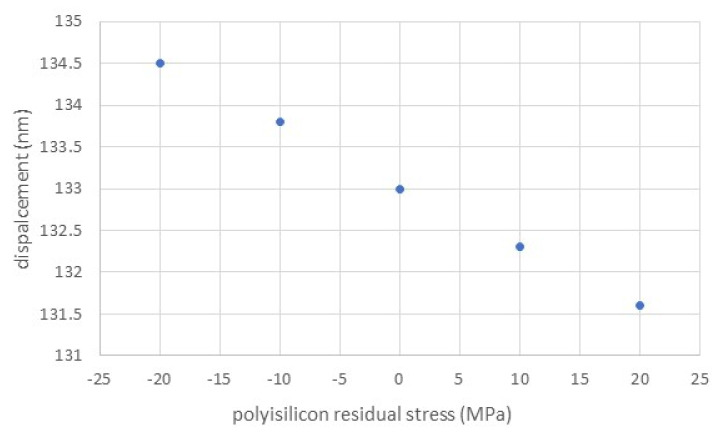
Maximum displacement as a function of polysilicon residual stress with a membrane side of 100 μm and a polysilicon thickness of 1.5 μm.

Given the different residual stress values reported in the literature for annealed polysilicon [20,21], the effect of possible stress fluctuations in the polysilicon layer was assessed by finite element simulation. Results are reported in Figure 5.

The displacement change within the explored stress range is 3 nm, indicating that this structure is not sensible to polysilicon stress variations caused by the polysilicon deposition and annealing process in a range from −20 to 20 MPa, which corresponds to the variations reported in the literature.

**Figure 6:**
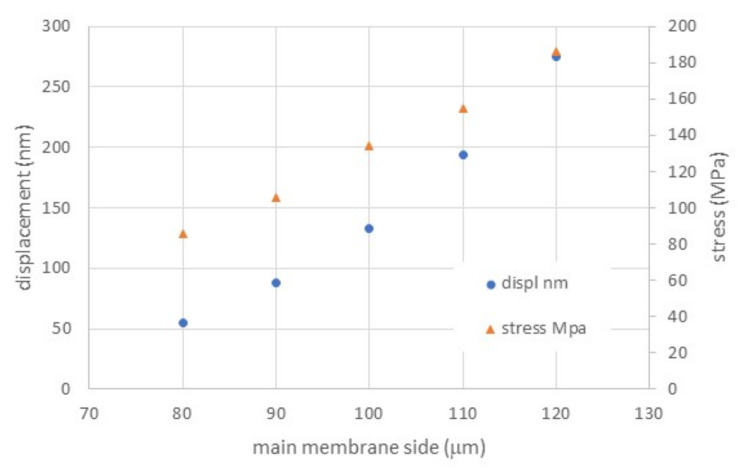
Maximum membrane displacement as a function of its side length, with polysilicon thickness of 1.5 μm.

Considering a possible uncertainty in TMAH etching in the dimensional control for such a small membrane size, the stress and displacement as a function of membrane side variation were also simulated, with results as reported in Figure 6.

A displacement from 55 to 275 nm was simulated when changing the membrane side from 80 to 120 μm. Though being a relatively large displacement variation, the membrane remains in a safe deformation regime, confirmed by its low stress values (Figure 6).

### 2.2. Experimental Setup and Orifice Fluid Dynamic Characterization

**Figure 7:**
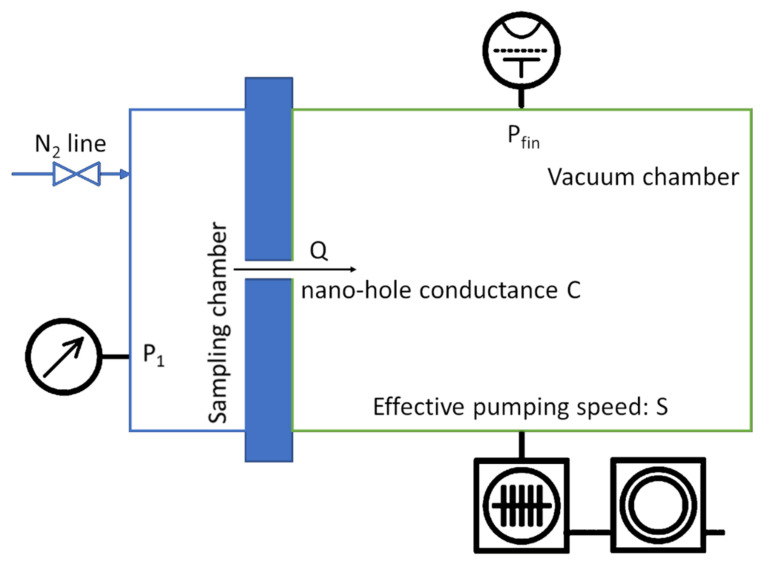
Schematic of the experimental setup. The sampling chamber (left) with a nitrogen inlet is connected to the vacuum chamber by the nanoscopic holes having a conductance C.

In order to qualify the geometry (i.e., diameters and depths) of the nano-fabricated orifices and to verify their performance in working conditions, a setup was realized to interface the fabricated membranes with a quadrupole mass filter spectrometer, operated at a background pressure ranging from 10^−9^ to 10^−10^ mbar. A schematic of the setup is reported in Figure 7.

**Figure 8:**
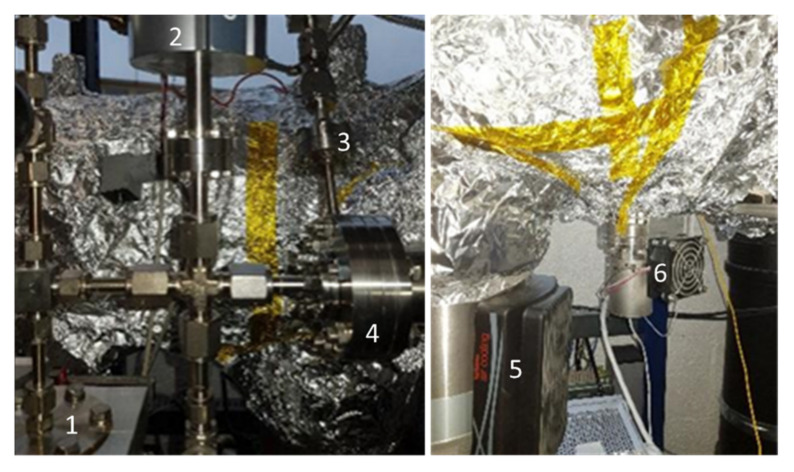
Membrane installation feature (1), pressure P_1_ and external gas line to be sampled (2), BA gauge for P_fin_ pressure (3), turbo-molecular pump (4), capacitive gauge for P_1_ (5), and P_fin_ on the membrane low-pressure side (6).

Figure 8 shows the main parts of the setup. The membrane chip is positioned across a pressure gradient between the high-pressure side (kept at pressures of 1013 mbar and above) and the low-pressure side (toward the quadrupole). The latter is pumped to extremely low background pressure by a turbo-molecular pump with a 400 L/s flow and by a rotary vane pump of 16 m^3^/h flow. Two measurement systems are installed to accurately measure the pressure on both membrane sides. A capacitance manometer (from 1 to 1000 Torr) is installed on the high-pressure side to precisely measure the sampling pressure of any gas or volatile species at a given pressure *P*_1_.

On the low-pressure side, a Bayard Alpert ionization gauge (BA) able to perform measurements to ~10^−11^ mbar is installed, together with a second capacitance manometer covering the range from 10^−1^ to 10^−4^ mbar, for calibration purposes. The system is equipped with pure nitrogen (99.999%) on the atmospheric pressure side, to avoid any potential influence coming from humidity, different gases, and the vapors mixture that demand additional calibration efforts and correction factors.

In the above test system, after degassing the low-vacuum side (i.e., the entire vacuum system including the MS) at 100 °C and calibrating the two vacuum gauges, a background pressure level *P*_0_ in the range of 10^−10^ mbar is obtained. Under these conditions, any N_2_ gas flow passing through the orifices at the given pressure *P*_1_ induces a pressure variation on the low-pressure side given by:

$$\Delta P=\;\left(P_{fin}-P_{0}\right)$$

where *P*_0_ is the background pressure and *P_fin_* is the measured pressure in the presence of a flow *Q* coming from the high-pressure region (*P*_1_) through the orifice. Thus, the inlet flow *Q* in the MS is:

(1) $$Q=\;\left(P_{fin}-P_{0}\right)\;*\;S$$

where *S* is the vacuum pump pumping speed for N_2_. On the other side, the amount of gas flow *Q* passing through the membrane is by definition:

(2) $$Q=\;\left(P_{1}-P_{fin}\right)\;*\;C$$

where *P*_1_ is the sampling pressure (normally in the range of ~1000 mbar) on the high-pressure side and *C* is the orifice conductance (L/s). As *P*_1_ is almost always at atmospheric level, then it is true that *P*_1_ >> *P_fin_*, due to the extremely low gas flow *Q* passing through nano-orifices (typically in the range from 10^−5^ to 10^−7^ mbar L/s). Therefore, Equation (2) can be simplified as follows:

(3) $$Q=\;P_{1}*C$$

Now, from the fabrication parameters of the realized orifices, we can assume that the orifice conductance is similar to that of a short tube whose dimensions *D* and *H* are respectively the diameter *D* of the tube and the height *H* of the tube [25]. In our case, *H* corresponds to the thickness of the fabricated sub-membranes, so the relation [25]:

(4) $$\frac{H}{D}\;<20$$

that guarantees the above approximation is always valid. Under these conditions, the conductance *C* of the single nano-orifice is [25]:

(5) $$C=C_{a}\;\frac{1}{\left(1+\frac{3}{4}*\frac{H}{D}\right)}$$

where *C_a_* is the conductance of the simple aperture of the single orifice with a diameter *D*. Then, in a molecular regime (thanks to the nanoscale dimensions), *C_a_* depends on the diameter *D* as per the following equation:

(6) Ca=π D24 * 1/(4 * 8RT⁄πM^1⁄2 

where *T*, *M*, and *R* are respectively the environment temperature, the molecular mass, and the universal constant of gas.

Finally, with a very low-pressure background, we can evaluate *Q* from Equation (1) by measuring *P*_1_ and *P_fin_*. Then, using Equations (3)–(6), we can calculate the conductance *C* from the slope of the curve Δ*P* vs. *P*_1_ and then *D* and *H* of each single orifice. In the molecular flow regime, this dependence is linear because the conductance *C* is normally independent of the pressure [25].

In this way, we can accurately measure the orifice geometry (*D* and *H*) from a fluid dynamics and operative standpoint.

## 3. Results

**Figure 9:**
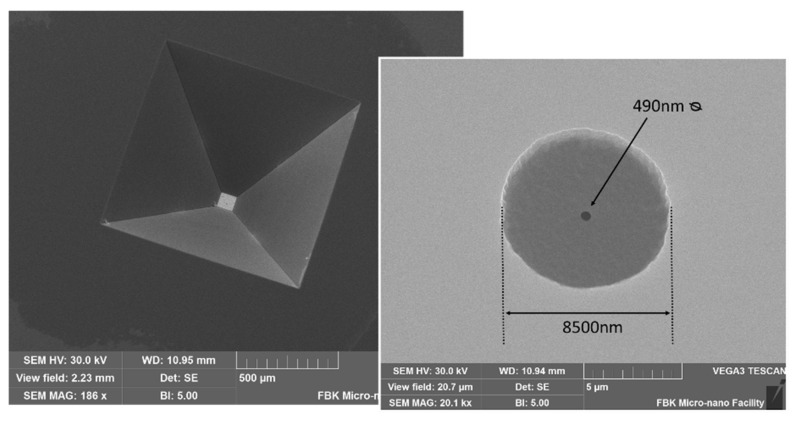
SEM image of a membrane from the chip backside (**left**) with detail of the sub-membrane and orifice (**right**).

A set of 30 membrane prototypes with nanoscopic holes was realized using the proposed microfabrication sequence in the microfabrication laboratory of FBK, Centre for Materials and Microsystems [26]. The hole diameters were in the range from 300 to 600 nm. The polysilicon thickness was 1.5 μm, and the sub-membrane (silicon-rich silicon nitride) thickness was 230 nm. The fabricated membrane side dimension was 84 μm with a standard deviation of 15 μm. The sub-membrane radius was 4 μm with a standard deviation of 1 μm. The chips were separated and bonded on perforated copper supports that provide a vacuum sealing interface. An example of the fabricated membranes is reported in Figure 9.

**Figure 10:**
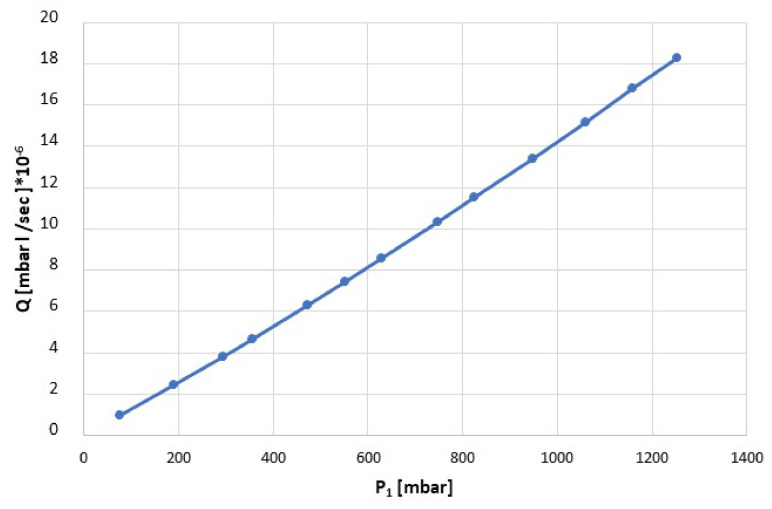
Flow measurement example of a membrane with 4 holes.

The chips were mounted in the above-described measurement setup, and flows were measured with nitrogen gas under high-vacuum conditions. In Figure 10, the flow measurement of a chip is reported as an example. The collected data of all measured chips were linear in the measured pressure range, with a fit having a square of correlation coefficient of 0.999.

## 4. Discussion

The mechanical performance of the fabricated membranes is in line with finite element simulation. During use, the chips were exposed to overpressures up to 1.8 bar and showed no mechanical damage. Further, after several vacuum cycles, there was no mechanical degradation: This was evident both from flow measurements and from SEM inspections after use. The very small membrane deflection under working conditions (few 100 nm with present design) is not only important to grant mechanical stability over time, but also intended to enable the future development of a MEMS valve to be fabricated on top of the membrane. The MEMS valve is necessary to perform fast switching between the open and closed state, which is now impossible due to dead volumes inherent with the use of macroscopic valves.

The diameter estimate by flow measurement is confirmed for some devices, with an accuracy of 10%. Others have an estimated diameter of up to 50% below the designed one. Measurements were repeated consistently on these anomalous devices, with a square of correlation coefficient of 0.999, and no cause was observed for this reduced diameter during SEM inspection. Further, no fault in the vacuum system can be the cause of this, as it would result in a larger estimated diameter. A possible cause may be resin solvent condensation on some chips during packaging, which could create a thin organic film on the silicon nitride. Further, the storing condition and environment may also cause degradation in the orifice performance. To study this phenomenon, the effect of post-packaging orifice cleaning procedures, such as oxygen plasma treatment both in vacuum and air, will be tested in future work.

Due to dust and contamination, the lifetime of this component could be critical and is expected to change depending on applications. All tests were performed using 450 nm Millipore^TM^ filters to avoid particles penetration into the system. The filters were replaced upon clogging. Moreover, in these preliminary tests, it was observed that devices tend to get clogged after repeated uses, and therefore, device replacement is needed: As reported, cleaning procedures are under study and will be included in future work. Storing and handling do not appear as critical steps in the performed experimental work: There is no evidence of a need for vacuum or nitrogen storing, as standard air with its dust does not compromise the stored chips.

Moreover, given the very small flows obtained with the proposed system, the signal in analytical applications may be reduced. To compensate this, a large number of holes can be implemented depending on the specific application: This can be obtained either by mounting several chips on the same frame, by fabricating more than one hole on each sub-membrane, or also by fabricating more sub-membranes on each membrane.

## 5. Conclusions

Microscale membranes with nanoscale holes were designed and fabricated using standard microelectromechanical techniques, to be used as inlets for novel analytical systems. A novel membrane-in-membrane approach was used to achieve stringent requirements of mechanical performance (1 bar pressure gradient) and orifice size. Microtechnology options were compared and discussed. Prototype devices were fabricated with this sequence, having a 230 nm-thick sub-membrane of silicon-rich silicon nitride, with orifice diameters from 300 to 600 nm.

Further, the devices were tested as inlets in an ultra-high-vacuum setup, demonstrating their mechanical properties and flow characteristics. A method to evaluate the effective holes diameter based on flow measurements was developed, obtaining a preliminary agreement on the hole diameter with respect to SEM measurements.

The reported prototypes demonstrate the feasibility of this technology in the framework of novel analytical systems development. The adopted materials and fabrication sequence grant wafer front side planarity, enabling very high accuracy patterning of the nanoscopic holes using conventional IC processing techniques, such as stepper lithography or e-beam lithography.

Future work will be addressed at enhancing the reproducibility of flow performance, by studying the effects of cleaning and storing conditions. Further, the possible development of a micromechanical valve to be fabricated on top of the membrane structure will be studied.

## Figures and Tables

**Table 1:** Silicon-rich silicon nitride properties and deposition rate as a function of deposition temperature.

Deposition Temperature (°C)	Refractive Index	Residual Stress (MPa)	Deposition Rate (nm/min)	Thickness Variation on Batch (%)
780	2.34	100	2.08	3
790	2.35	41	2.13	5
795	2.37	14	2.48	11

## Data Availability

Data are contained within the article.

## Appendix A. Fabrication Procedure

With reference to Figure 2, below are the fabrication process details. All LPCVD and annealing processes were performed with a Centrotherm E1200HT (Blaubeuren, Germany). All photolithography exposure was performed with a Nikon 2205i11D (Tokyo, Japan).

Figure 2a: Silicon nitride barrier deposition by LPCVD at 720 °C, thickness 52 nm (st.dev. 4 nm), followed by LPCVD TEOS (tetraethoxysilane glass) deposition at 630 °C, thickness 590 nm (st.dev. 8 nm), and patterned using stepper photolithography with a Fujifilm OIR674 photoresist and dry etching with a TEGAL 900 (Tegal Corporation, Petaluma, CA, USA) plasma etcher.

Figure 2b: Polysilicon is deposited by PECVD at 620 °C with a thickness of 1480 nm (st.dev. 11 nm), and recrystallized by annealing at 1050 °C for 30 min in nitrogen atmosphere. LPCVD silicon-rich silicon nitride is then deposited at 790 °C with a thickness of 230 nm (st.dev. 3 nm). Details of this deposition are reported in the Materials and Methods section.

Figure 2c: Nanoscale orifices are patterned in the silicon nitride layer, using stepper photolithography with a Fujifilm OIR674 photoresist, and Alcatel SMS 200 (SPTS Technologies, Newport, UK) dry etching. A second LPCVD TEOS (tetraethoxysilane glass) is deposited at 630 °C, with a target thickness of 600 nm (no thickness measurement was performed).

Figure 2d: The wafer backside mask is patterned using stepper photolithography with a Fujifilm OIR674 photoresist and dry etching with a TEGAL900 (Tegal Corporation, Petaluma, CA, USA) plasma etcher. Anisotropic silicon etching is performed in a quartz reactor using tetramethylammonium hydroxide (TMAH) at 25 wt.% concentration and 82 °C temperature for 18 h.

Figure 2e: The silicon nitride is removed from the membrane backside by wet etching in an 85 wt.% H_3_PO_4_ solution at 140 °C. A second TMAH etching is performed at 26 wt.% concentration and 82 °C temperature for 4 min to release the sub-membrane. TEOS is removed in 8 wt.% HF solution for 10 min.
